# Autophagy Enhances Bacterial Clearance during *P. aeruginosa* Lung Infection

**DOI:** 10.1371/journal.pone.0072263

**Published:** 2013-08-28

**Authors:** Robert D. Junkins, Ann Shen, Kirill Rosen, Craig McCormick, Tong-Jun Lin

**Affiliations:** 1 Department of Microbiology and Immunology, Dalhousie University, Halifax, Nova Scotia, Canada; 2 Department of Pediatrics, IWK Health Centre, Halifax, Nova Scotia, Canada; 3 Beatrice Hunter Cancer Research Institute, Halifax, Nova Scotia, Canada; 4 Department of Biochemistry and Molecular Biology, Halifax, Nova Scotia, Canada; Louisiana State University, United States of America

## Abstract

*Pseudomonas aeruginosa* is an opportunistic bacterial pathogen which is the leading cause of morbidity and mortality among cystic fibrosis patients. Although *P. aeruginosa* is primarily considered an extacellular pathogen, recent reports have demonstrated that throughout the course of infection the bacterium acquires the ability to enter and reside within host cells. Normally intracellular pathogens are cleared through a process called autophagy which sequesters and degrades portions of the cytosol, including invading bacteria. However the role of autophagy in host defense against *P. aeruginosa in vivo* remains unknown. Understanding the role of autophagy during *P. aeruginosa* infection is of particular importance as mutations leading to cystic fibrosis have recently been shown to cause a blockade in the autophagy pathway, which could increase susceptibility to infection. Here we demonstrate that *P. aeruginosa* induces autophagy in mast cells, which have been recognized as sentinels in the host defense against bacterial infection. We further demonstrate that inhibition of autophagy through pharmacological means or protein knockdown inhibits clearance of intracellular *P. aeruginosa in vitro*, while pharmacologic induction of autophagy significantly increased bacterial clearance. Finally we find that pharmacological manipulation of autophagy *in vivo* effectively regulates bacterial clearance of *P. aeruginosa* from the lung. Together our results demonstrate that autophagy is required for an effective immune response against *P. aeruginosa* infection *in vivo*, and suggest that pharmacological interventions targeting the autophagy pathway could have considerable therapeutic potential in the treatment of *P. aeruginosa* lung infection.

## Introduction

*Pseudomonas aeruginosa* is an environmentally ubiquitous gram negative bacterial pathogen which is a leading cause of morbidity and mortality among cystic fibrosis (CF) patients and the immunocomprimised [1]. In healthy individuals *P. aeruginosa* infection triggers strong inflammatory responses, mediated largely through TLR signaling pathways, which lead to neutrophil recruitment and effective clearance of the bacteria [2]. The coordination of these early host responses to the pathogen are largely mediated by resident immune cells in the airway such as mast cells or alveolar macrophages [3], [4], [5]. Mast cells are recognized as sentinel cells of the immune system in the respiratory tract where they represent up to 2% of the alveolar wall and protrude into the airspace of the lung where they are ideally placed to be first responders to invading pathogens [6]. Upon encountering pathogens mast cells not only produce various cytokines to coordinate further immune responses [7], [8], but also act as phagocytes, internalizing and killing invading organisms [9]. Active interactions between mast cells and *P. aeruginosa* have been observed [8], [10], [11].

CF patients almost invariably become chronically infected with *P. aeruginosa.* Lung infection with *P. aeruginosa* correlates clinically with decreased lung function and impaired survival [12]. Many factors contribute to the increased susceptibility to *P. aeruginosa* infection observed in CF patients. Mutations in the cystic fibrosis transmembrane conductance receptor (CFTR) which cause CF lead to osmotic dysregulation resulting the accumulation of thick mucus at the surface of epithelial cells which impairs the clearance of pathogens from the lungs [13]. Furthermore CFTR mutations have been shown to dysregulate TLR signaling and surface expression leading to impaired and prolonged inflammatory responses to the pathogen [2]. However, recently a novel effect of mutations to the CFTR has been identified which leads to dysregulation of an evolutionarily conserved catabolic process called macroautophagy, which is hereafter referred to as autophagy [14]. Mutations to the CFTR have been shown to lead to upregulation of reactive oxygen species (ROS) production, and enhanced tissue transglutaminase activity which combine to drive the crosslinking and inactivation of the beclin-1 PI3K complex which represents a central component of the autophagy pathway [14].

Autophagy is an evolutionarily conserved catabolic process through which portions of the cytosol are sequestered and degraded within highly specialized double membrane bound vesicles termed autophagosomes. Over the past decade autophagy has emerged as a central component of the innate and adaptive immune responses where it plays roles in antigen presentation including cross-presentation, direct and indirect killing of intracellular and extracellular pathogens, generation of bactericidal peptides and the regulation of inflammatory responses [15], [16], [17]. Autophagy has been implicated in *P. aeruginosa* infection in cultured macrophages *in vitro* [18]. However, the biological significance of autophagy in *P. aeruginosa* infection *in vivo* and its role in mast cell-*P. aeruginosa* interaction remain undefined.

One of the greatest challenges in the treatment of *P. aeruginosa* infection is the highly antibiotic resistant nature of the bacteria [19]. The recent emergence of multi-drug resistant *P. aeruginosa* strains leading to increased morbidity and mortality in susceptible populations highlights the need for novel therapeutic strategies for the treatment of *P. aeruginosa* infections [20], [21], [22]. Recently it has been proposed that *P. aeruginosa* bacteria have the ability to reside within host cells where they can evade host immune cells, and that the development of intracellular infections may represent a mechanism contributing to antibiotic resistance [23], [24]. Given the well characterized central role of autophagy in the clearance of intracellular pathogens [25], and the observation that autophagy is impaired in the airways of cystic fibrosis patients, we set out to examine the role of autophagy in host defense against *P. aeruginosa in vivo*, and explored the therapeutic potential of pharmacological manipulation of the autophagy pathway during *P. aeruginosa* lung infection. Our results demonstrate that *P. aeruginosa* infection induces autophagy in mast cells which are abundant in the airways where they play a central role in host defense against *P. aeruginosa* [5], [7], as well as in bronchial epithelial cells which have been proposed to act as a reservoir of intracellular bacteria during chronic *P. aeruginosa* infection [24], [26], [27], [28], [29]. We further demonstrated that inhibition of the autophagy pathway significantly impairs clearance of *P. aeruginosa* from mast cells and human bronchial epithelial cells, while induction of the process enhances bacterial killing. Finally we demonstrate that pharmacological manipulation of the pathway effectively regulates bacterial clearance *in vivo*. Thus, induction of autophagy could represent a novel therapeutic approach for the treatment of *P. aeruginosa* infection.

## Materials and Methods

### Ethics statement

All animal protocols were approved by the University Committee on Laboratory Animals, Dalhousie University, in accordance with the guidelines of the Canadian Council on Animal Care. Animals were housed in specific pathogen free facilities, and anesthetized with ketamine to minimize suffering during relevant procedures.

### Animals

C57/BL6 mice were purchased from Charles River Laboratories (Wilmington, MA) and used between the ages of 8–10 weeks.

### Antibodies

Antibodies for actin (sc1616), GFP (sc8334), mouse mast cell tryptase (mMCP6) (sc32473), rabbit anti-goat IgG HRPO (sc2768), donkey anti-mouse IgG HRPO (sc2314), goat anti-rabbit IgG HRPO (sc2004) and biotin conjugated mouse anti-goat IgG (sc2489) were purchased from Santa Cruz Biotechnology (Dallas, TX). An antibody for LC3 (clone 2G6) was purchased from Nano Tools (San Diego, CA). Antibodies for Atg5 (#2630) and Atg7 (#2631) were purchased from Cell Signaling Technology (Danvers, MA).

### Bacterial internalization and killing

Bacterial internalization and killing was measured as previously described [8], [30]. Briefly, 1×10^5^ wild-type HMC-1 5C6 cells, or 1×10^4^ 16HBE14o^−^ or CFBE41o^−^ cells were left untreated or pretreated for 1 hour with 20 µM chloroquine diphosphate salt (Simga Aldrich, St. Louis, MO) or 2 µM rapamycin (LC Laboratories, Woburn, MA). Alternatively, untreated HMC-1 5C6 cells stably expressing Atg5 or Atg7 shRNA were used. Cells were infected in 100 µL of serum free IMDM media with *P. aeruginosa* strain 8821 at a 1∶20 MOI for 3 hours. Extracellular bacteria were then killed with cell impermeable antibiotics (50 U/mL each of penicillin and streptomycin (Life Technologies, Burlington, ON), 200 µg/mL gentamycin (Life Technologies), 100 µg/mL ceftazidime hydrate (Sigma Aldrich) and 100 µg/mL piperacillin sodium salt (Sigma Aldrich)) for 10 minutes (for the study of bacterial internalization) or 3 hours (for the study of bacterial killing). Cells were then washed in PBS, and lysed in 100 µL PBS containing 0.2% Triton X-100 (Sigma Aldrich) and a serial dilution of 10 µL of lysate was streaked in duplicate on LB agar plates, and incubated overnight at 37°C. CFUs were counted the next day. Samples containing no cells were used to identify background CFUs which were subtracted from samples at each time point.

### Bacterial preparation

The laboratory strains of *P. aeruginosa* PAK and PAO.1 were gifts from Dr. J. Boyd (Institute for Marine Bioscience, National Research Council, Halifax, NS) and Dr. D. Wozniak (Ohio State University, Columbus, OHIO, USA) respectively. *P. aeruginosa* strain 8821, a mucoid strain isolated from a cystic fibrosis patient, was a gift from Dr A. Chakrabarty (University of Illinois, Chicago, IL, USA) [31]. *P. aeruginosa* strains were cultured as described previously [32]. Briefly, suspension cultures were grown until reaching the early stationery phase. Bacteria were washed in phosphate buffer and resuspended in 0.9% saline for *in vivo* experiments or PBS for *in vitro* assays. Mice were infected as described below. *Escherichia coli* stain DH5α was purchased from Life Technologies (Burlington, ON) and cultured according to the suppliers instructions.

### Cell Culture

Mouse bone marrow-derived mast cells (BMMCs) were cultured from C57/BL6 mice as previously described [33]. Mast cells were confirmed by toluidine blue staining and following 5–6 weeks in culture, mast cell purity was >98%.

Human mast cells HMC-1 5C6 were a kind gift from Dr. Dr. B. M. Henz (Virchow Klinikum, Berlin) [34] and were maintained in IMDM supplemented with 10% FBS and 50 U/mL each of penicillin and streptomycin in a 5% CO_2_-humidified atmosphere at 37°C. Culture medium was further supplemented with 500 µg/mL G418 for the maintenance of HMC-1 5C6 cell stably expressing LC3-GFP, and 10 µg/mL puromycin for the maintenance of HMC-1 5C6 cells stably expressing NT, Atg5 or Atg7 shRNA. All experiments were carried out in the absence of selection.

Highly purified cord blood-derived mast cells (CBMC) (>95% purity) were obtained by long term culture of cord blood progenitor cells as previously described [9]. The percentage of mast cells in the cultures was determined by toluidine blue staining of cytocentrifuged samples. Mature mast cells after more than 8 weeks in culture were identified by their morphological features and the presence of metachromatic granules, at which time they were used for this study.

The human epithelial cell line 16HBE14o^−^ and the stable CFTR ΔF508 homozygous cell line CFBE41o^−^ were gifts from Dr. D. Gruenert (University of California, San Francisco) and have been described previously [35], [36]. Cells were maintained in MEM supplemented with 10% FBS and 50 U/mL each of penicillin and streptomycin in a 5% CO_2_-humidified atmosphere at 37°C.

### Cytokine production

Concentrations of IL-1β, TNF, IL-6, MIP2 and RANTES in BALF and lung supernatants was determined by ELISA as described previously using antibody pairs from R&D Systems (Minneapolis, MN, USA) [8].

### LC3-GFP assay

Two million HMC-1 5C6 cells stably expressing LC3-GFP were left untreated or treated as indicated. Cells were then fixed at various time points in 4% paraformaldehyde and cytocentrifuged onto glass slides for 5 minutes at 200 rpm for examination by fluorescence microscopy (Nikon Eclipse E600; Nikon, Tokyo, Japan) and by confocal laser scanning microscopy (Zeiss LSM510, Zeiss, Toronto, Ontario, Canada). Alternatively 16HBE14o^−^ cells were grown to confluence on poly-L-lysine coated glass coverslips and transiently transfected with LC3-GFP using Lipofectamine 2000 (Life Technologies) according to the manufacturer's instructions. Cells were then left untreated, or treated as indicated and fixed in 4% paraformaldehyde and examined as described above. At least one hundred cells per slide were counted and cells containing 5 or more GFP punctuations were considered LC3 positive.

### Lung infection with P. aeruginosa and collection of lung and bronchioalveolar lavage fluid (BALF)

Mice were infected with 1×10^9^ CFU *P. aeruginosa* intranasally for 24 hours. BALF was then obtained by lavaging the lung with 3× 1 mL PBS containing 100 µg/mL soybean trypsin inhibitor (Sigma Aldrich). The lung tissues were obtained for detection of cytokines, myeloperoxidase (MPO) and bacterial colony-forming units (CFU) counting.

Lung tissues were homogenized in 50 mM HEPES buffer (4 µL/1 mg lung) containing 100 µg/mL soybean trypsin inhibitor. For counting bacterial CFU, 10 µL of the homogenate was plated onto an agar dish and incubated for 24 h at 37°C. The homogenate was centrifuged at 4°C for 30 min at 18000 g. The supernatant was stored at −80°C for later cytokine analysis. The pellet was resuspended and homogenized in 0.5% cetyltrimethylammonium chloride (CTAC) (4 µL/mg lung) and centrifuged as above. The cleared extract was used for MPO assay.

BALF (10 µL) was plated on an agar dish and incubated for 24 h for CFU counting. For detection of cytokines and MPO activity, BALF was centrifuged at 480 g for 5 minutes at 4°C. The supernatants were used for cytokine analysis. The pellets were resuspended in 1 mL NH_4_Cl (0.15 M) and centrifuged as before to lyse red blood cells. The supernatants were discarded and the pellets were resuspended in 0.5% CTAC (250 µL/mouse) then centrifuged. The cleared extracts were used for MPO assay.

For survival studies, mice were infected with 1×10^9^ CFU *P. aeruginosa* strain 8821 as described above. Mice were then monitored for 10 days, and moribund animals were euthanized.

### Mast cell nucleofection and generation of stable cell lines

HMC-1 5C6 cell were nucleofected with LC3-GFP, LC3-mCherry, NT shRNA, Atg5 shRNA or Atg7 shRNA as previously described [37]. Briefly HMC-1 5C6 cells were resuspended at 4×10^6^ cells/transfection in Amaxa nucleofector solution and electroporated with 8 µg DNA using Amaxa Nucleofector II Device program U-023 (Lonza, Basel, Switzerland).

HMC-1 5C6 cells stably expressing LC3-GFP were generated by culturing nucleofected cells under 500 µg/mL G418 (Invitrogen) selection for 4 weeks starting 24 hours after nucleofection. Cells were then sorted for GFP^mid^ expressing cells using a BD FACSAria1 (BD Biosciences, Mississauga, Ontario), and maintained under G418 selection. HMC-1 5C6 cells stably expressing NT, Atg5 or Atg7 shRNA were generated by culturing nucleofected cells under 10 µg/mL puromycin selection for 4 weeks starting 24 hours after nucleofection. Knockdown was confirmed by Western blot analysis as described below.

### Mast cell tryptase (mMCP6) solid phase ELISA

Mice were left untreated, or infected with *P. aeruginosa* strain 8821 as described above. Lung tissue homogenates, BALF supernatants and serum proteins were absorbed onto a high binding Nunc MaxiSorp 96 well plate (Thermo Fischer Scientific, Rochester, NY) overnight at 4°C. Plates were washed 4 times with 200 μL 0.01% Tween-20 in PBS then blocked for 2 hours at room temperature in 2% BSA in PBS. Plates were washed 4 times with 200 μL 0.01% Tween-20 in PBS then anti-mouse tyrptase antibody diluted 1∶50 in 0.2% BSA, 0.05% Tween-20 in PBS was added and incubated overnight at 4°C. Plates were washed 4 times with 200 μL 0.01% Tween-20 in PBS then biotin conjugated mouse anti-goat IgG diluted 1∶100 in 0.2% BSA, 0.05% Tween-20 in PBS was added and incubated at room temperature for 2 hours. Plates were washed 5 times with 200 μL 0.01% Tween-20 in PBS then ELISAs were developed using the Invitrogen ELISA amplification system (Life Technologies) according to the manufacturer's directions.

### MPO assay

The MPO assay was used to determine the infiltration of neutrophils into the lungs of the mice as described previously [38]. Briefly, samples in duplicate (75 µL) were mixed with equal volumes of the substrate (3,3′,5,5′-tetramethyl-benzidine dihydrochloride, 3 mM; Resorcinol, 120 µM; and H_2_O_2_, 2.2 mM) for 2 minutes. The reaction was stopped by adding 150 µL of 2 M H_2_SO_4_. The optical density was measured at 450 nm.

### Pharmacological manipulation of autophagy in vivo

Autophagy was inhibited *in vivo* through the administration of chloroquine diphosphate salt (Sigma Aldrich, St. Louis MO) intraperitoneally in PBS at a dose of 60 mg/kg/day for 3 days prior to infection as described previously [39]. Autophagy was induced *in vivo* through the administration of rapamycin (LC Laboratories, Woburn, MA) in diluent (5.2% Tween 80, 5.2% polyethylene glycol 400 in sterile water) at a dose of 10 mg/kg, 24 hours prior to infection as described previously [40].

### Plasmids

LC3-GFP was generated as previously described [41] and was generously donated by Dr. T. Yoshimori (Osaka University, Japan). LC3-mCherry was generated as previously described [42] and was generously donated by Dr. T. Johansen (University of Tromso, Norway). Non-targeting, Atg5 and Atg7 specific shRNA were generated as previously described [43].

### Statistics

Data are presented as mean ± SEM of the indicated number of experiments. Statistical significance between treatment groups was determined using ANOVA and post-hoc Tukey's honest significance multiple comparisons test, or when there where only two groups are being compared, by an unpaired t-test. Statistical analysis was performed using GraphPad Prism 5 (GraphPad Software Inc., La Jolla, CA).

### Transmission Electron Microscopy

Untreated and *P. aeruginosa* treated mast cells were fixed in 2.5% glutaraldehyde, postfixed in 1% osmium tetroxide, dehydrated with ethanol, and embedded in epoxy resin for thin sectioning, followed by standard staining in uranium and lead salts, as described previously [44]. Thin sections were observed in a JEOL JEM-1230 transmission electron microscope equipped with a Hamamatsu ORCA-HR high-resolution (2,000 by 2,000 pixels) digital camera, and images were saved as TIFF files. Autophagosomes were identified based on the appearance of their characteristic double membrane, and heterogeneous contents (Reviewed in [45]). For area analysis electron micrographs were saved as TIFF images so that 1 pixel is representative of 1 unit area of the cell. The area of the cytoplasm was then defined using Photoshop (Adobe, San Jose, CA) by subtracting the number of pixels contained within the nuclear envelope from the number of pixels contained within the plasma membrane. The number of pixels in each cell contained within autophagosomes was then determined and expressed as a percentage of the number of pixels contained within the cytoplasm of the cell.

### Western Blot and Scanning Densitometry

Cells lysates (15–40 µg) were subjected to electrophoresis in 10% or 12% SDS-polyacrylamide gels. Gels were transferred to polyvinylidene difluoride membrane, blotted with primary and secondary antibodies as indicated, and detected by an enhanced chemiluminescence detection system (Western Lightning Plus-ECL; PerkinElmer, Waltham, MA). Scanning densitometry analysis was performed using Scion Image (Scion Corporation, Frederick, MD).

## Results

### P. aeruginosa induces autophagy in mast cells

**Figure 1:**
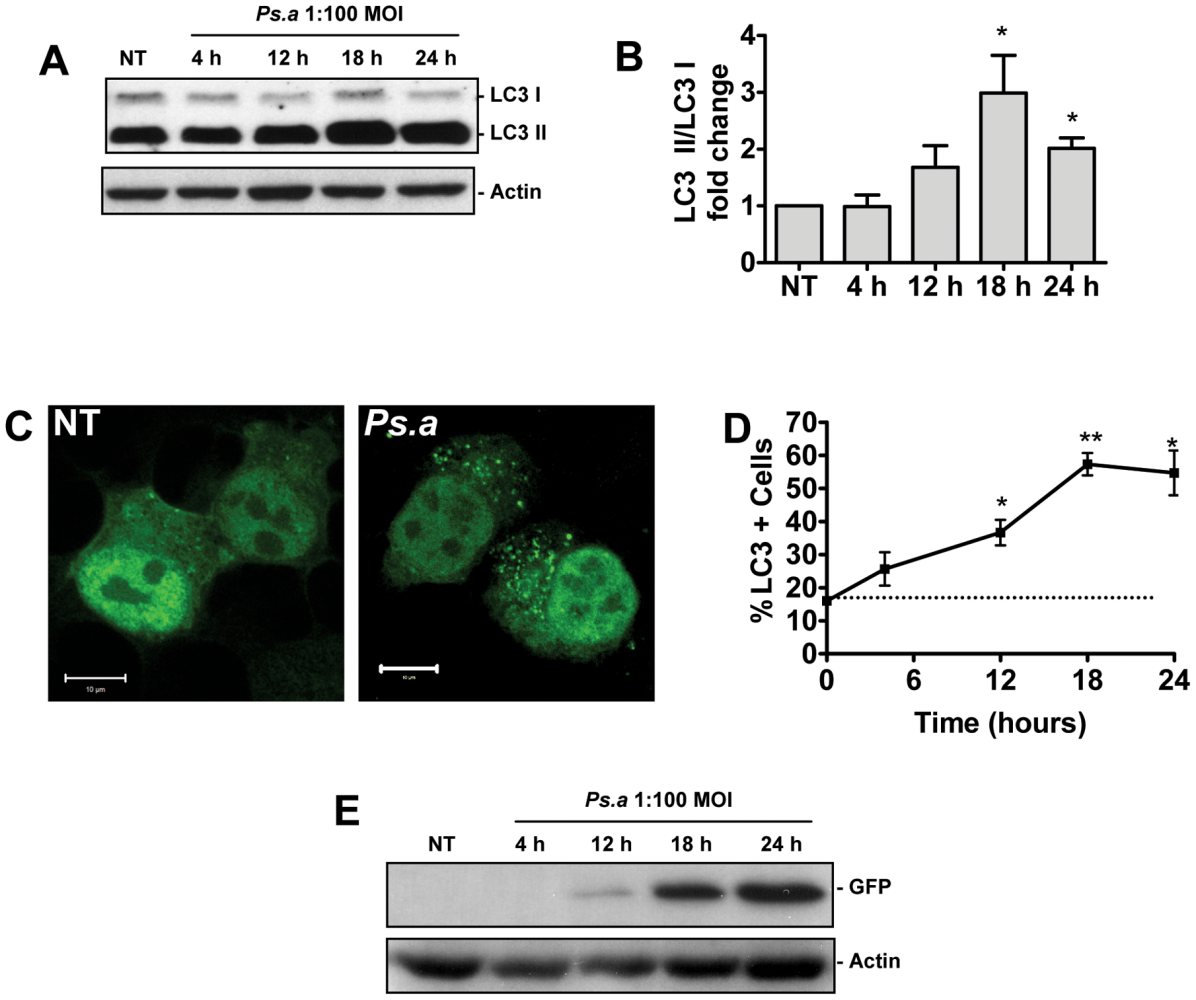
*P. aeruginosa* induces autophagy in mast cells. Primary mouse BMMCs were left untreated (NT) or infected with *P. aeruginosa* strain 8821 at an MOI of 1∶100. Lysates were collected at the time points indicated and subjected to Western blot analysis for LC3 and actin loading control (A). The fold change in the ratio of LC3-I to LC3-II normalized to actin was determined by scanning densitometry (B) (n = 3 ± SEM, *p<0.05). HMC-1 5C6 human mast cell line stably expressing LC3-GFP reporter were left untreated (NT) or infected with *P. aeruginosa* at an MOI of 1∶100 for 18 hours (*Ps.a*) before being fixed and examined by laser scanning confocal microscopy (C). HMC-1 5C6 cells stably expressing LC3-GFP were left untreated (NT) or infected with *P. aeruginosa* strain 8821 at an MOI of 1∶100. At the indicated time points cells were fixed for the study by confocal microscopy, and the percentage of cells displaying 5 or more GFP puncta was assessed (D). (n = 3± SEM, *p<0.05, **p<0.01). Lysates were also prepared from HMC-1 5C6 cells stably expressing LC3-GFP and Western blot analysis for free GFP and actin loading control was performed (E).

Mast cells are important sentinel cells of the immune system, playing a critical role in sensing invading pathogens and coordinating the appropriate immune response against *P. aeruginosa* [5], [7]. Due to the high density of the cells along the airways, and their phagocyte capacity, mast cells also represent the first line of defense against pathogens within the respiratory tract. Our lab has extensively studied the roles of mast cells during host defense against *P. aeruginosa*. However the role of autophagy in mast cells in the context of host defense, as well as the biological role of autophagy during *P. aeruginosa* infections remains unknown. In order to examine the role of autophagy in mast cells during *P. aeruginosa* infection, bone marrow derived mast cells (BMMCs) were cultured from C57/BL6 mice. These cells were then infected with *P. aeruginosa* strain 8821 at an MOI of 1∶100. Whole cell lysates were prepared at various time points as indicated, and subjected to western blot analysis for microtubule associated protein light chain 3 (LC3) and actin loading control. Upon the induction of autophagy the cytoplasmic form of LC3 (LC3-I) becomes cleaved and conjugated to phophotidylethanolamine (PE) through a ubiquitin like conjugation pathway. This PE conjugated form of the protein (LC3-II) becomes and remains associated with autophagosomal membrane throughout the maturation cycle of the vesicle. The conversion of cytosolic LC3-I to autophagosome associated LC3-II is diagnostic of autophagy and can be tracked by Western blot analysis [46]. In untreated cells, the PE conjugated LC3-II form of the protein predominated within the cells, consistent with a previously describe role for LC3 in mast cell granule formation [47]. However while the cytoplasmic LC3-I levels remained unchanged upon stimulation with *P. aeruginosa,* the levels of LC3-II accumulated well above basal levels, peaking around 18 hours post infection (hpi), indicating an induction of autophagy (Figure 1A and 1B).

Autophagy can also be monitored using a construct consisting of LC3 conjugated to GFP. Upon the induction of autophagy, LC3-GFP becomes redistributed from a diffuse cytosolic distribution to distinct GFP positive puncta representing autophagosomes. In order to monitor autophagy following *P. aeruginosa* infection using this technique, the HMC-1 5C6 human mast cell line was stably transfected with an LC3-GFP construct. These cells were then infected with *P. aeruginosa* strain 8821 at a 1∶100 MOI and fixed at various time points post-infection for examination by fluorescence microscopy. The untreated cells displayed primarily a diffuse cytosolic distribution of LC3-GFP prior to exposure to *P. aeruginosa*. However upon stimulation with the bacteria for 18 hours the fluorescence became largely localized to discrete GFP positive puncta indicative of autophagosomes (Figure 1C). In order to further quantify the induction of autophagy, the percentage of cells at each time point which displayed greater than 5 discrete GFP puncta was assessed (Figure 1D). A time dependent increase in GFP puncta positive cells was observed, which peaked 18 hpi, and was significantly increased compared to untreated cells between the 12 and 24 hpi time points.

HMC-1 5C6 cells stably expressing LC3-GFP were further used to assess flux through the autophagy pathway. As autophagosomes mature fluorescently inactive GFP is cleaved from LC3-II and accumulates in the cell. The appearance of this free GFP can be tracked as a measure of flux through the autophagy pathway [48], [49], [50]. LC3-GFP expressing HMC-1 5C6 cells were left untreated (NT) or infected with *P. aeruginosa* at an MOI of 1∶100. Lysates were prepared at the time points indicated, and were subjected to Western blot analysis for free GFP (Figure 1E). No free GFP was detected in untreated cells. However free GFP protein began to appear in the cells as soon as 12 hpi, and continued to increase at longer time points. These data demonstrate that autophagy is induced in mast cells following *P. aeruginosa* infection, and exposure to the bacteria promotes flux through the pathway.

We further characterized the dose response of autophagy induced by *P. aeruginosa* strain 8821, PAK and PAO.1 as well as *E. coli* stain DH5α. A dose-dependent increase in LC3-GFP puncta formation in HMC-1 5C6 cells was observed in response to all strains of bacteria suggesting that the induction of autophagy in mast cells may be a generalized host response to *P. aeruginosa* and other gram negative bacteria (Figure S1).

As previous reports have suggested that LC3 becomes incorporated into the membranes of mast cell granules [47], we set out to ensure the LC3 positive structures we observed were not mast cell granules. Because the fixation conditions required for staining mast cell granules are acidic, the HMC-1 5C6 LC3-GFP cell line could not be used to examine colocalization of LC3 positive puncta with mast cell granules as GFP has a pKa of 6.0 and is not fluorescent under acidic conditions [51], [52]. In order to visualize LC3 localization under these conditions HMC-1 5C6 cells were transiently transfected with LC3-mCherry, which retains fluorescence under acidic pH [52]. LC3-mCherry expressing cells were then fixed and stained for mast cell granules using toluidine blue and subjected to fluorescent and light microscopy (Figure S2A). Consistent with previous reports, the HMC-1 5C6 cells were not well granulated, with only 21% of cells containing granules [53]. Of the granulated cells there was on average only 7 granules per cell. The number of mast cell granules and the number of LC3-mCherry positive mast cell granules were counted in 100 cells containing at least one granule, and one LC3-mCherry positive puncta (Figure S2B). Colocalization between LC3 and mast cell granules was very rarely observed. Furthermore, there was no correlation between the number of LC3-mCherry positive puncta, and the number of mast cell granules in a given cell (Figure S2C). Together these results indicate that LC3 positive structures in HMC-1 5C6 cells are not mast cell granules.

### P. aeruginosa induces autophagy in primary human and mouse mast cells and becomes incorporated into autophagosomes

**Figure 2:**
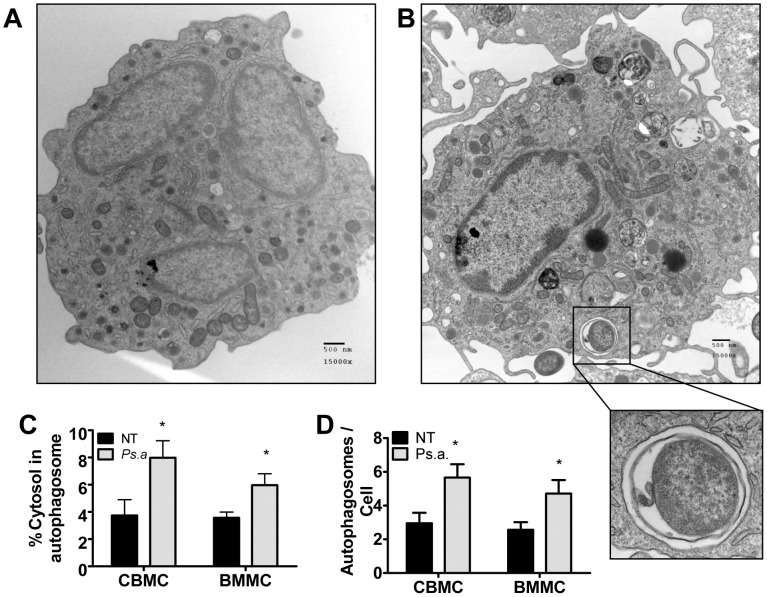
*P. aeruginosa* induces autophagy in primary human and mouse mast cells, and becomes incorporated into autophagosomes. Primary human cord blood derived mast cells (CBMCs) and mouse bone marrow-derived mast cells (BMMCs) were left untreated (NT) or infected with *P. aeruginosa* strain 8821 at an MOI of 1∶100 for 8 hours. Cells were then fixed and processed for transmission electron microscopic study. Representative images of untreated (A) and *P. aeruginosa* treated (B) CBMCs are shown. *P. aeruginosa* could clearly be seen inside double membrane bound vesicles inside the mast cells (insert). The percentage of cytosol encompassed within autophagosomes (C) and the number of autophagosomes per cross section (D) in CBMCs and BMMCs was calculated by area analysis of cross sections containing a portion of the cell nucleus (n = 21± SEM, *p<0.05).

We next set out to examine the ultrastructural characteristics of autophagosomes in primary human and murine mast cells. To address this question we infected primary human cord blood derived mast cells (CBMCs) and primary mouse BMMCs with *P. aeruginosa* strain 8821 at an MOI of 1∶100. Eight hours later cells were fixed and processed for TEM viewing (Figure 2A and 2B). Highly vesicularized double membrane bound vesicles characteristic of autophagosomes were observed in both untreated (NT) and *P. aeruginosa* (Ps.a) treated cells. The percentage of cytosol contained within autophagosomes, as well as the number of autophagosomes per cross-section was significantly increased in *P. aeruginosa* treated cells (Figure 2C and 2D). Furthermore, *P. aeruginosa* bacteria were repeatedly seen inside the cell contained within double membrane bound vesicles (insert), although these observations were infrequent. Together these results demonstrate that autophagy is induced by *P. aeruginosa* in primary human and mouse mast cells, and that the bacteria can become incorporated into autophagosomes *in vitro*.

### Autophagy contributes to bacterial killing by mast cells in vitro following P. aeruginosa infection

**Figure 3:**
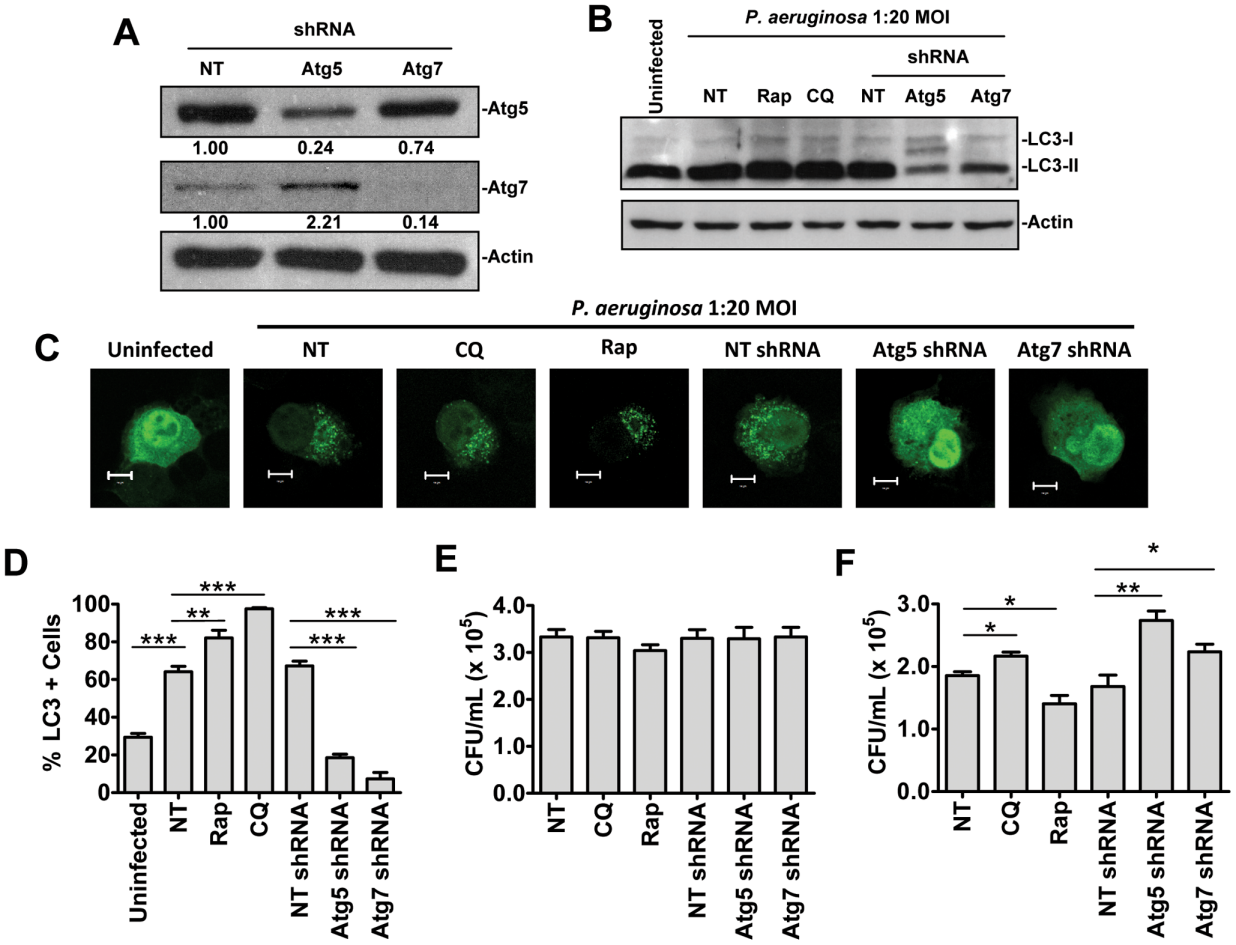
Autophagy contributes to bacterial killing by mast cells *in vitro* following *P. aeruginosa* infection. HMC-1 5C6 cells stably expressing non-targeting (NT) shRNA or shRNA specific for the autophagy genes Atg5 or Atg7 were subjected to western blot analysis to confirm protein knockdown (A). Numbers below each band represent fold change vs. NT shRNA group and normalized to actin as determined by scanning densitometry. Wild-type HMC-1 5C6 cells were left untreated (NT) or pretreated for one hour with 20 µM chloroquine (CQ) or 2 µM rapamycin (Rap). These cells, along with HMC-1 5C6 cells stably expressing NT, Atg5 or Atg7 specific shRNA were left uninfected, or infected at a 1∶20 MOI with *P. aeruginosa* strain 8821 for 3 hours. Cell impermeable antibiotics were added to kill extracellular bacteria for 3 hours. Cells were then lysed and subjected to Western blot analysis for the autophagy marker LC3 and acting loading control (B). Alternatively HMC1-5C6 cells expressing LC3-GFP and treated as above were monitored by laser scanning confocal microscopy (C and D) (n = 3 ± SEM, **p<0.01, ***p<0.005). Lysates were prepared from HMC-1 5C6 cells treated as described above, and incubated in the presence of cell impermeable antibiotic for 10 minutes to determine bacterial internalization (E) or 3 hours to examine bacterial killing (F). Serial dilutions of these lysates were streaked on LB agar plates and CFU/mL lysate was determined (n = 6± SEM, *p<0.05, **p<0.01).

Having observed bacteria inside autophagosome like structures, we next set out to examine the impact of autophagy on bacterial killing in mast cells following *P. aeruginosa* infection. In order to examine the role of autophagy in bacterial killing HMC-1 5C6 cell lines stably expressing non-targeting shRNA (HMC-1 NT shRNA) or shRNA directed against the essential autophagy genes Atg5 (HMC-1 Atg5 shRNA) or Atg7 (HMC-1 Atg7 shRNA) were generated. Knockdown was confirmed by western blot analysis (Figure 3A). Knockdown efficiency for Atg5 and Atg7 shRNA were determined to be 76% and 86% respectively as determined by scanning densitometry.

In order to assess the role of autophagy in the killing of internalized bacteria wild-type HMC-1 cells were left untreated (NT), or pretreated for one hour with the autophagy inhibitor chloroquine, or the autophagy inducer rapamycin. These cells, along with HMC-1 cells expressing non-targeting (NT), or Atg5 or Atg7 specific shRNA, were left uninfected or infected for 3 hours at a 1∶20 MOI with *P. aeruginosa* strain 8821. Cells were then treated with cell impermeable antibiotics for 3 hours to kill extracellular bacteria. The effect of each treatment on autophagy was assessed by LC3 Western blot analysis (Figure 3B) and using an LC3-GFP assay (Figures 3C and 3D). Autophagy was induced following *P. aeruginosa* infection, and a further accumulation of autophagosome associated LC3-II was observed when autophagic flux was blocked by chloroquine, or when autophagy was pharmacologically induced with rapamycin. Knockdown of Atg5 and Atg7 significantly decreased autophagy compared to NT shRNA and wild-type controls. These treatments were then used to assess the impact of autophagy on bacterial internalization and killing in cultured mast cells. No significant differences were observed when cells were treated with cell impermeable antibiotics for 10 minutes indicating that genetic or pharmacological manipulation of the autophagy pathway did not affect internalization of the bacteria (Figure 3E). Importantly, in the bacterial killing assay where cells were treated with cell impermeable antibiotics for 3 hours, manipulation of the autophagy pathway was observed to differentially regulate the killing of internalized bacteria (Figure 3F). Inhibition of autophagy through pharmacological and genetic means resulted in significantly decreased bacterial killing compared to untreated, and non-targeting shRNA controls. Conversely, induction of autophagy with rapamycin was found to significantly increase bacterial killing following *P. aeruginosa* infection. These results suggest that autophagy contributes to the killing of internalized bacteria in mast cells, and pharmacological manipulation of the pathway can enhance clearance of intracellular *P. aeruginosa.*

### Pharmacological manipulation of autophagy modulates the clearance of P. aeruginosa from bronchial epithelial cells

**Figure 4:**
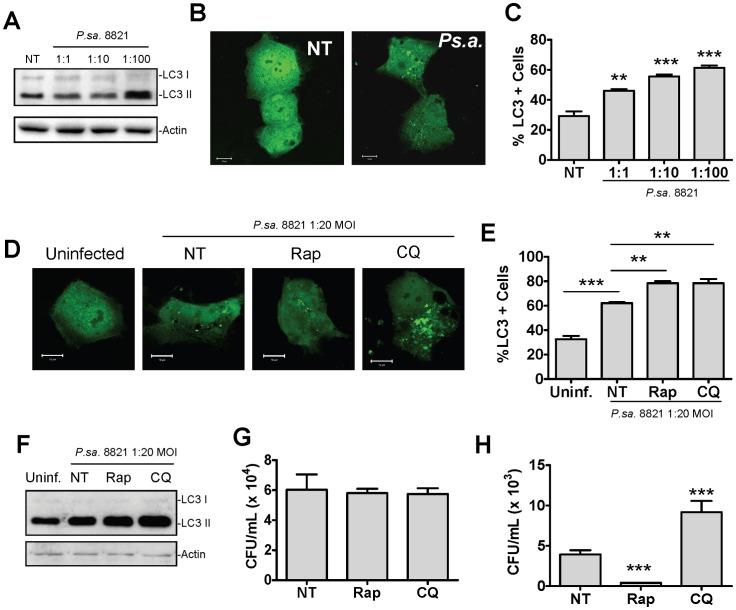
Autophagy enhances clearance of intracellular *P. aeruginosa* from normal human bronchial epithelial cells. The human bronchial epithelial cell line 16HBE14o^−^ was left uninfected, or infected with *P. aeruginosa* at an MOI of 1∶1, 1∶10 and 1∶100. Lysates were collected 18 hours post infection and subjected to Western blot analysis for LC3 and actin (A). Alternatively, cells were transiently transfected with LC3-GFP two days prior to infection then treated as described above. Eighteen hours post infection cells were fixed and examined by laser scanning confocal microscopy (B) and the percentage of cells containing greater than 5 LC3-GFP puncta was determined (C) (n = 3± SEM, **p<0.01, ***p<0.005). Untransfected, or LC3-GFP expressing 16HBE14o^−^ cells were left untreated, or pretreated for one hour with rapamycin (2 μM) or chloroquine (20 μM) then infected at a 1∶20 MOI with *P. aeruginosa* strain 8821. Cell impermeable antibiotics were added 3 hours later, then after an additional 3 hour incubation cells were either fixed for evaluation of autophagy by confocal microscopy (D and E) (n = 3± SEM, *p<0.05, **p<0.01, ***p<0.005) or lysed and analyzed by Western blot analysis for LC3 and actin (F). Alternatively, cells were lysed 10 minutes (G) or 3 hours (H) after the addition of cell impermeable antibiotics and streaked on LB agar plates for the determination of intracellular CFUs (n = 5± SEM, ***p<0.005). The 10 minute and 3 hour time points represent bacterial internalization and killing respectively.

Given the ability of *P. aeruginosa* to enter and reside within bronchial epithelial cells [24], [26], [27], [28], [29], we set out to examine the role of autophagy in these cells during *P. aeruginosa* infection. The human bronchial epithelial cell line 16HBE14o^−^ was left untreated or was infected with *P. aeruginosa* strain 8821 at MOIs of 1∶1, 1∶10 and 1∶100. Eighteen hours post infection lysates were collected and subjected to Western blot analysis for LC3 and actin (Figure 4A). Alternatively, 16HBE14o^−^ cells transiently transfected with LC3-GFP were treated as above, and fixed for examination by confocal microscopy (Figure 4B and 4C). Autophagy was found to be induced by *P. aeruginosa* in these cells in a dose dependent manner indicating that the induction of autophagy within the airways by *P. aeruginosa* is not restricted to mast cells.

We next assessed whether manipulation of autophagy in these cells could modulate clearance of intracellular bacterial as was observed in mast cells. Human bronchial epithelial cells (16HBE14o^−^ cells) were either transiently transfected with LC3-GFP for confocal microscopy or were left untransfected for Western blot analysis. Cells were then either left untreated or were pre-treated for one hour with chloroquine to inhibit autophagic flux, or rapamycin to induce autophagy. Cells were left uninfected or infected with *P. aeruginosa* at an MOI of 1∶20 for 3 hours after which cell impermeable antibiotics were added for an additional 3 hours. Finally cells were either fixed for confocal microscopy (Figure 4D and 4E) or lysed for Western blot analysis (Figure 4F). Treatment with either chloroquine or rapamycin did not affect the internalization of *P. aeruginosa* (Figure 4G). As was observed in mast cells, chloroquine treatment inhibited bacterial killing (with increased number of intracellular bacteria). In contrast, treatment with rapamycin promoted bacterial killing (with reduced bacterial numbers in 16HBE14o^−^ epithelial cells) (Figure 4H).

In order to assess the therapeutic potential of these treatments in a model of CF, internalization and killing of *P. aerginosa* under the above conditions was further compared in normal human 16HBE14o^−^ bronchial epithelial cells, and CFTR ΔF508 homozygous CFBE41o^−^ epithelial cells. Consistent with previous reports, cells harboring CFTR ΔF508 mutations displayed decreased internalization of *P. aeruginosa* (Figure S3A) [28]. Interestingly, CFBE41o^−^ cells also displayed a marked impairment in their ability to kill internalized bacteria. Pharmacological manipulation of autophagy differentially regulated killing of internalized bacteria in these cells. Induction of autophagy with rapamycin promoted bacterial killing (Figure S3B). These results suggest that pharmacological manipulation of autophagy not only enhances clearance of *P. aeruginosa* by normal epithelial cells, but can also restore bacterial clearance from epithelial cells harboring mutations in CFTR.

### Pharmacological manipulation of autophagy differentially regulates bacterial clearance in vivo following P. aeruginosa infection

Having identified that pharmacological manipulation of autophagy regulates killing of *P. aeruginosa* bacteria by mast cells and bronchial epithelial cells *in vitro* we next set out to study the therapeutic potential of pharmacological manipulation of autophagy *in vivo* during *P. aeruginosa* lung infection. However since the contribution of mast cells during *P. aeruginosa* infection *in vivo* is incompletely understood, we first examined whether mast cells are activated during *P. aeruginosa* infection *in vivo*. The mast cell specific protease mMCP-6 was used as mast cell activation marker *in vivo*. The level of mMCP-6 in the serum, BALF and lung was measured in mice 24 hours after infection with *P. aeruginosa* strain 8821 (1×10^9^ CFU/mouse). *P. aeruginosa* infection increased levels of mMCP-6 in the lungs and serum (Figure S4). These data suggest that mast cells are activated during *P. aeruginosa* lung infection.

**Figure 5:**
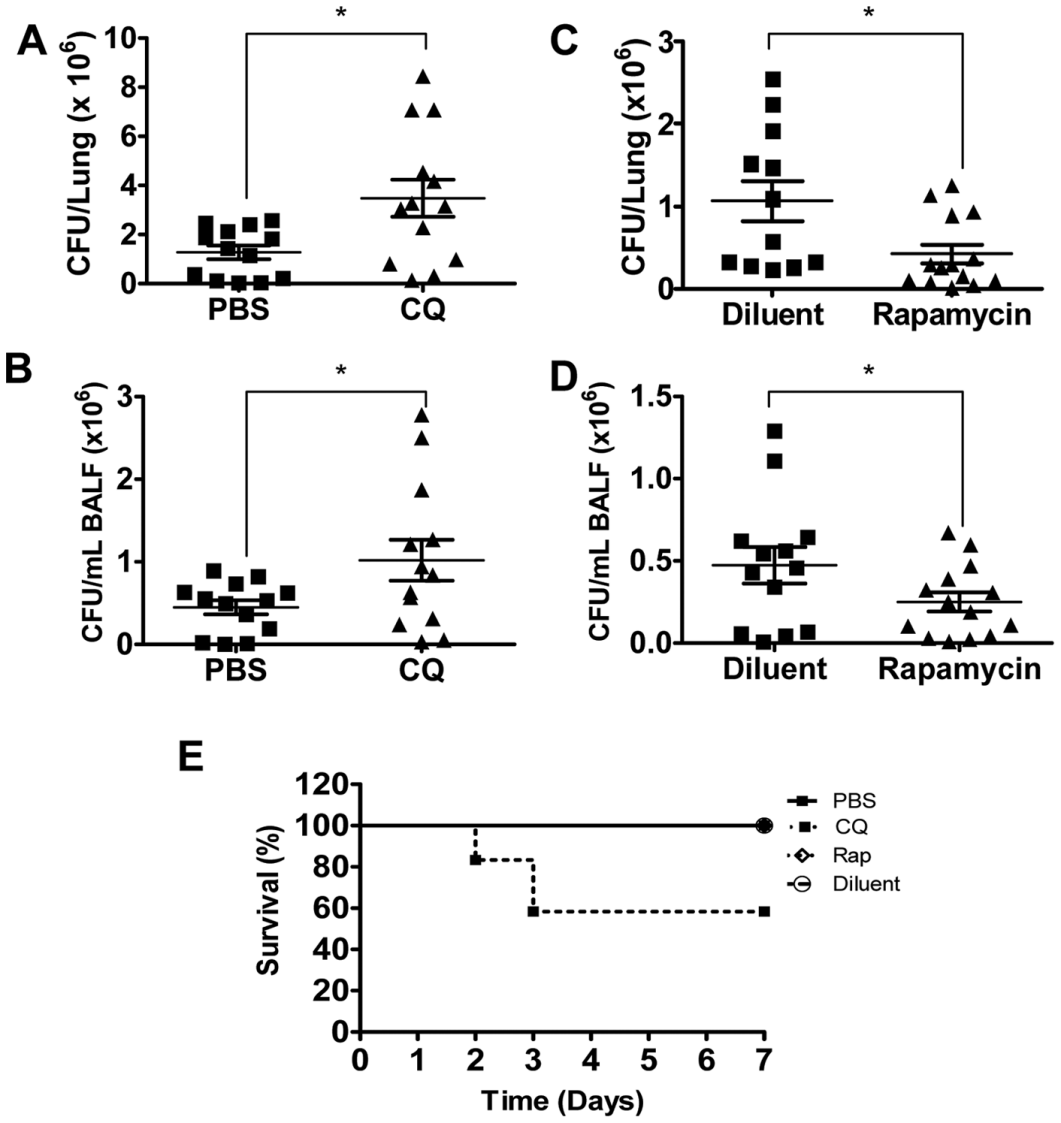
Pharmacological manipulation of autophagy modulates *P. aeruginosa* clearance *in vivo*. C57BL/6 mice were treated with intraperitoneal injection of PBS or 60 mg/kg/day chloroquine (CQ) in PBS for 3 days (A–B, E) or with intraperitoneal injection of diluent or 10 mg/kg/day rapamycin for 1 day (C–E), then infected intranasally with 10^9^ CFU/mouse *P. aeruginosa* strain 8821. Twenty four hours later mice were sacrificed and CFU was counted in the Lung (A, C) and BALF (B, D). (n = 13–15± SEM,*p<0.05). Alternatively animal survival was monitored for 10 days post infection (n = 15± SEM, *p<0.05).

In order to assess the impact of inhibition of autophagy on *P. aeruginosa* lung infection, autophagy inhibitor chloroquine was used. *In vitro* experiments demonstrated that chloroquine did not impact mast cell numbers or viability during *P. aeruginosa* infection (Figure S5). Mice were pretreated with CQ by intraperitoneal injection at a dose of 60 mg/kg/day for 3 days prior to infection, or received an equivalent volume of PBS. Mice were then challenged intranasally with *P. aeruginosa* strain 8821 or a saline control. Twenty four hours later mice were sacrificed and lung tissue and bronchioalveolar lavage fluid (BALF) was collected. Bacterial burden was assessed by counting CFUs in serial dilutions of lung homogenates (Figure 5A) and BALF (Figure 5B). CQ treatment significantly increased bacterial load following *P. aeruginosa* infection in both the lungs and the BALF. Animal survival was also assessed for 10 days post infection with 1×10^9^ CFU *P. aeruginosa* strain 8821. While no mortality was observed in animals treated with PBS control, 40% mortality was observed in the chloroquine treated mice (n = 15 mice/group, p<0.05) (Figure 5E). Thus, treatment with CQ reduced the clearance of *P. aeruginosa* from the lung and impaired animal survival.

Given that neutrophil recruitment to the site of infection contributes to the clearance of *P. aeruginosa*, we assessed neutrophil accumulation in the lungs and BALF of CQ and saline treated mice through assaying the activity of the neutrophil specific enzyme myeloperoxidase (MPO). MPO activity was unaffected by CQ treatment both in the lungs and the BALF (Figure S6A). The autophagy pathway has also been proposed to play a role in regulating inflammatory cytokine production [54]. In order to determine whether the differences in bacterial clearance observed in chloroquine treated mice were associated with dysregulation of cytokine responses, the levels of various inflammatory cytokines were assessed in the lungs and BALF of *P. aeruginosa* and saline treated mice pretreated with PBS or chloroquine (Table S1). No significant differences were observed in the levels of any of the cytokines assayed suggesting that manipulation of the autophagy pathway did not impact host inflammatory responses. Together these data suggest that the defect in bacterial clearance in CQ treated mice is associated not with coordination of the immune response, but instead with impaired bacterial killing.

Having demonstrated that pharmacological disruption of the autophagy pathway impairs host defense against *P. aeruginosa*, we next set out to test the therapeutic potential of pharmacological induction of the pathway. One of the best studied pharmacological inducers of autophagy is rapamycin, which promotes autophagy through inhibition of the mammalian target of rapamycin (mTOR), a master regulator of the autophagy pathway [55], [56]. Similar to chloroquine treatment, rapamycin treatment did not impact mast cell numbers or viability during *P. aeruginosa* infection *in vitro* (Figure S5). Mice were pretreated with rapamycin at a dose of 10 mg/kg, or an equivalent volume of diluent both at the time of infection, and one day prior. Mice were infected intranasally with 1×10^9^ CFU/mouse with *P. aeruginosa*. Twenty four hours later mice were sacrificed and lung tissue and BALF were collected. Bacterial burden was assessed by counting CFUs in serial dilutions of lung homogenates (Figure 5C) and BALF (Figure 5D). We found that rapamycin significantly reduced the bacterial load in both the lungs and the BALF of rapamycin treated mice compared to diluent treated control mice. To examine whether pharmacological modulation of autophagy affects animal survival, mice were pretreated with rapamycin or diluent and intranasally infected with *P. aeruginosa* strain 8821. Animal survival was observed for 7 days. *P. aeruginosa* did not cause mortality in mice treated rapamycin or diluent (Figure 5E).

We next assessed cytokine production (Table S2) and neutrophil infiltration (Figure S6B) in the lungs and BALF of rapamycin and diluent treated mice. Consistent with the reported anti-inflammatory roles of rapamycin, treatment of mice with rapamycin showed significantly reduced levels of almost every inflammatory cytokine tested [57]. As a result, neutrophil recruitment into the lungs and BALF of rapamycin treated animals was also impaired. Together these data suggest that in spite of the impaired inflammatory responses observed in rapamycin treated animals, the accompanying induction of autophagy was able to enhance bacterial clearance above the levels observed in diluent treated animals, supporting a critical role for autophagy in the clearance of *P. aeruginosa* bacteria *in vivo*.

## Discussion

*P. aeruginosa* infection remains the number one cause of morbidity and mortality among cystic fibrosis patients who almost invariably become chronically infected with the bacteria [12]. Autophagy represents an evolutionarily conserved mechanism for the clearance of intracellular pathogens, and recent reports have shown the pathway to be dysregulated in the lungs of cystic fibrosis patients [14]. In the present study we examined the contribution of autophagy to the clearance of the cystic fibrosis pathogen *P. aeruginosa*. We found that *P. aeruginosa* induces autophagy in mast cells, which play an important role as sentinel cells during *P. aeruginosa* lung infection. Furthermore bacteria were observed inside autophagosomes, and pharmacological or genetic manipulation of the pathway modulated clearance of internalized bacteria *in vitro*. Similarly pharmacological modulation of autophagy also modulated clearance of *P. aeruginosa* from human epithelial cells. Induction of autophagy using rapamcyin was also able to correct defects in the clearance of intracellular bacteria observed in epithelial cells harboring CFTR ΔF508 mutations. Finally, pharmacological manipulation of the autophagy pathway effectively regulated bacterial clearance from the lungs of infected mice *in vivo*. Together these findings suggest that autophagy is induced in mast cells and epithelial cells in response to *P. aeruginosa.* Pharmacological manipulation of autophagy has considerable therapeutic potential for the treatment of *P. aeruginosa* lung infection.

The emergence of multi-antibiotic resistant strains of *P. aeruginosa* represents a very real threat to the life expectancy and quality of life in cystic fibrosis patients [20], [21], [22]. One possible contributing factor to the antibiotic resistant nature of *P. aeruginosa* is the observation that the bacteria has the ability infect host cells, where it can survive for long periods of times within the cytosol, sheltered from cell impermeable antibiotics [24], [26], [27], [28], [29]. Normally, intracellular pathogens are targeted for degradation through the autophagy pathway [15]. However, the autophagy pathway has been shown to be impaired in cystic fibrosis patients by mutations in CFTR which lead to dysregulation of the beclin-1 PI3K complex [14]. Given our observations that pharmacological manipulation of the autophagy pathway *in vivo* effectively regulates the clearance of a strain of *P. aeruginosa* isolated from a cystic fibrosis patient, and that pharmacological induction of autophagy corrects defects in the clearance of intracellular bacteria from CF epithelial cells, it is highly likely that impaired autophagy in cystic fibrosis patients contributes to colonization with the bacteria. As a result, therapies targeted at restoring or enhancing normal autophagy in the lungs of cystic fibrosis patients could significantly enhance clearance of the bacteria from lungs. Therapies aimed at restoring normal autophagy in cystic fibrosis patients have recently been reviewed [58], and treatment of mice harboring mutations in the CFTR with the autophagy inducer rapamycin has been shown to enhance clearance of the cystic fibrosis pathogen *Burkhoderia cenocepacia* from the lungs [40]. Together with our data, these findings demonstrate the potential of autophagy based therapy in treating common infections in cystic fibrosis patients. Promisingly, autophagy based therapies could serve as an excellent compliment to conventional antibiotic therapies. While antibiotic therapies can target extracellular populations of bacteria, the induction of autophagy has the ability to target intracellular bacteria. Targeting both populations could not only enhance killing of the bacteria, but also eradicate a pool of bacteria contributing to chronic infections, and the development of antibiotic resistance [24]. Furthermore, restoring normal autophagy in mice harboring mutations in CFTR also resolves chronic inflammation observed in the lungs [59], suggesting that as well as resolving *P. aeruginosa* infection, combining antibiotic therapy with autophagy therapy could help resolve damaging inflammation associated with deteriorating lung function in cystic fibrosis patients [60], [61].

Therapeutic manipulation of the autophagy pathway has become a topic of considerable interest over the past decade due to implication of the pathway in a wide range of pathologies. However, despite the recent interest in autophagy modulators, the inducers of autophagy currently available surfer from considerable undesirable off target effects, raising the need for development of novel, specific autophagy inducers. Current autophagy inducers can be grouped into three kinds of compounds: i) mTOR inhibitors, ii) modulators of calcium dependant signaling or iii) IP_3_ inhibitors [61]. Each of pathways targets plays integral roles in multiple cell pathways making them poor therapeutic targets for the specific induction of autophagy. In the current study, the mTOR inhibitor rapamycin was used to induce autophagy. In addition to inducing autophagy, rapamycin also has well characterized immunosuppressive effects [57]. Accordingly, decreased inflammatory cytokine production was observed in our model which led to impaired neutrophil recruitment to the site of infection. These defects were likely independent of the effects of rapamycin on autophagy as inhibition of the pathway did not impact inflammatory cytokine production. Similarly, chloroquine, the autophagy inhibitor used in this study, is a lystoropic base which raises the pH of lysosomes, preventing degradation through the autophagy pathway. However inhibition of classical phagocytic killing by chloroquine cannot be ruled out. It is important to note that in spite of the immunosuppressive effects of rapamycin which led to decreased neutrophil recruitment to the lungs of infected mice, enhanced bacterial clearance was observed following rapamycin treatment, reinforcing the contribution of autophagy to bacterial killing in our model. The development of specific autophagy modulators, as well as therapeutic strategies for restoring normal autophagy in cystic fibrosis patients will be an essential step towards improved treatment of *P. aeruginosa* infection. Excitingly, recent work has identified a peptide fragment from beclin-1 which strongly and specifically induces autophagy, and was able to decrease mortality associated with viral pathogens *in vivo* [62]. Such targeted therapeutic strategies for the modulation of autophagy have considerable clinical potential for the effective control and irradiation of *P. aeruginosa* lung infections.

Intracellular pathogens have developed a wide range of mechanisms for subverting or usurping the host autophagy pathway to promote their pathogenicity and survival [63], [64]. Given the novel observations that autophagy contributes to the clearance of *P. aeruginosa in vivo*, it raises the possibility that the bacteria have evolved similar mechanisms to avoid killing through the autophagy pathway. *P. aeruginosa* bacteria employs various mechanisms which skew the host immune response towards a Th2 phenotype [65], [66]. It has long been thought that a Th2 like response is favorable in the clearance of extracellular infections, while a Th1 response favors killing of intracellular infections. However, in the case of *P. aeruginosa* infections a Th2-like response is in fact correlated with poorer lung function and clinical outcomes compared to the minority of patients which mount a Th1-like response, underscoring the importance of the intracellular phase of *P. aeruginosa* infection [67], [68], [69]. Interestingly, Th2 cytokines have been demonstrated to suppress autophagy, suggesting that the Th2 responses driven by *P. aeruginosa* could promote the bacteria's intracellular survival by preventing activation of the autophagy pathway. In addition, *P. aeruginosa* secretes exotoxin A which shuts down host protein synthesis through inhibiting eukaryotic elongation factor 2A (eEF2A) which greatly enhances the virulence of the bacteria [70]. However disruption of the eEF2A signaling pathways has also been shown to impair activation of autophagy [71]. Thus, *P. aeruginosa* may employ multiple mechanisms for disrupting the autophagy pathway, contributing to enhanced bacterial survival and pathogenicity.

Given the increasing prevalence of multi-antibiotic resistant strains of *P. aeruginosa* bacteria, novel therapeutic approaches for the treatment of chronic infections in individuals with cystic fibrosis will be essential to ensure the continued health of these patients in the coming years. Here we demonstrate that *P. aeruginosa* induces autophagy in mast cells, and that the pathway contributes to the killing of internalized bacteria *in vitro*. We further demonstrate that therapeutic intervention aimed at inducing autophagy with rapamycin correlates with decreased bacterial loads following *P. aeruginosa* lung infection *in vivo,* in spite of decreased inflammatory responses and neutrophil infiltration caused by rapamycin treatment. This work provides the first evidence that the pharmacological manipulation of the autophagy pathway has considerable therapeutic potential in the treatment of *P. aeruginosa* infections *in vivo*.

## Supporting Information

Figure S1 **LC3 does not colocalize with granules in HMC-1 cells.** HMC-1 5C6 cells were transiently transfected with LC3-GFP-mCherry then fixed and stained with toluidine blue 48 hours later. Cells were examined by fluorescence and light microscopy then LC3-mCherry positive puncta, and toluidine blue positive granules were identified. Colocalization of LC3 puncta (indicated with arrows) with mast cell granules was assessed (A). The average number of total granules and LC3 positive granules per cell was determined (B) (n = 100± SEM, ***p<0.005). The correlation between the number of granules and the number of LC3 positive puncta in each cell was also assessed (C) (n = 100).
(TIF)

Figure S2 **Strain and dose effects of** ***P. aeruginosa*** **and** ***E. coli*** **on autophagy in mast cells.** Wild-type or LC3-GFP expressing HMC-1 5C6 cells were left untreated or infected at a 1∶1, 1∶10 and 1∶100 MOI with *P. aeruginosa* strains 8821 (A, E), PAO.1 (B, F), PAK (C, G) or *E. coli* strain DH5α (D, H). Cells were incubated for 18 hours at 37°C then lysed for Western blot analysis of LC3 and actin (A–D) or fixed and examined by fluorescence microscopy for the percentage of cells containing greater than 5 LC3-GFP puncta (E-H) (n = 3± SEM, *p<0.05, **p<0.01, ***p<0.005).
(TIF)

Figure S3 **Induction of autophagy restores bacterial clearance in CF epithelial cells.** Ten thousand 16HBE14o- (HBE) normal human bronchial epithelial cells or CFBE41o- (CFBE) homozygous CFTR ΔF508 cystic fibrosis bronchial epithelial cells were left untreated (NT) or pretreated for one hour with 20 µM chloroquine (CQ) or 2 µM rapamycin (Rap). Cells were then infected at a 1∶20 MOI with *P. aeruginosa* strain 8821 for 3 hours. Cell impermeable antibiotics were added for 10 minutes (A) or 3 hours (B) then serial dilutions of cell lysates were streaked to assess intracellular CFUs. The 10 minute and 3 hour time points represent bacterial internalization and bacterial killing respectively.
(TIF)

Figure S4 **Mast cells contribute to host defense against** ***P. aeruginosa.*** C57BL/6 mice were left uninfected, or infected intranasally with 109 *P. aeruginosa* strain 8821. Twenty four hours later serum, BALF and lung tissue was collected and the relative concentrations of the mast cell specific protease mMCP6 were determined by solid phase ELISA (n = 6 ± SEM, *p<0.05).
(TIF)

Figure S5 **Pharmacological manipulation of autophagy does not effect mast cell survival following** ***P. aeruginosa*** **infection.** BMMCs from C57BL/6 mice were pretreated for 24 hours with 20 µM chloroquine (CQ), 2 µM rapamycin (Rap), or an equivalent volume of PBS or rapamycin diluent. Cells were then infected with *P. aeruginosa* strain 8821 at an MOI of 1∶10 for 24 hours after which cell density (A) and viability (B) was determined by trypan blue staining (n = 3± SEM).
(TIF)

Figure S6 **Rapamycin but not chloroquine impairs neutrophil infiltration into the lungs and BALF during** ***P. aeruginosa*** **lung infection.** C57BL/6 mice were treated with intraparteneal injections of PBS or 60 mg/kg/day chloroquine (CQ) in PBS for 3 days (A) or with intraparteneal injections of diluent or 10 mg/kg/day rapamycin for 1 day (B), then infected intranasally with 109 CFU/mouse *P. aeruginosa* strain 8821. Twenty four hours later mice were sacrificed and lung tissue and BALF was assayed for the activity of the neutrophil specific enzyme MPO (n = 13-15± SEM, *p<0.05).
(TIF)

Table S1 C57BL/6 mice were treated with intraperitoneal injections of PBS or 60 mg/kg/day chloroquine (CQ) in PBS for 3 days, and then left uninfected, or infected intranasally with 10^9^ CFU/mouse *P. aeruginosa* strain 8821. Twenty four hours later mice were sacrificed and lung tissue and BALF was collected and assayed for concentrations of indicated cytokines via ELISA.
(DOC)

Table S2 C57BL/6 mice were treated with intraperitoneal injections of diluent or 10 mg/kg/day rapamycin for 1 day, and then left uninfected, or infected intranasally with 10^9^ CFU/mouse *P. aeruginosa* strain 8821. Twenty four hours later mice were sacrificed and lung tissue and BALF was collected and assayed for concentrations of indicated cytokines via ELISA.
(DOC)
