# Supplementary figures and images for: Autophagy Enhances Bacterial Clearance during P. aeruginosa Lung Infection
Source: PLoS One. 2013 Aug 28;8(8):e72263. doi: 10.1371/journal.pone.0072263 (PMC3756076; doi:10.1371/journal.pone.0072263)

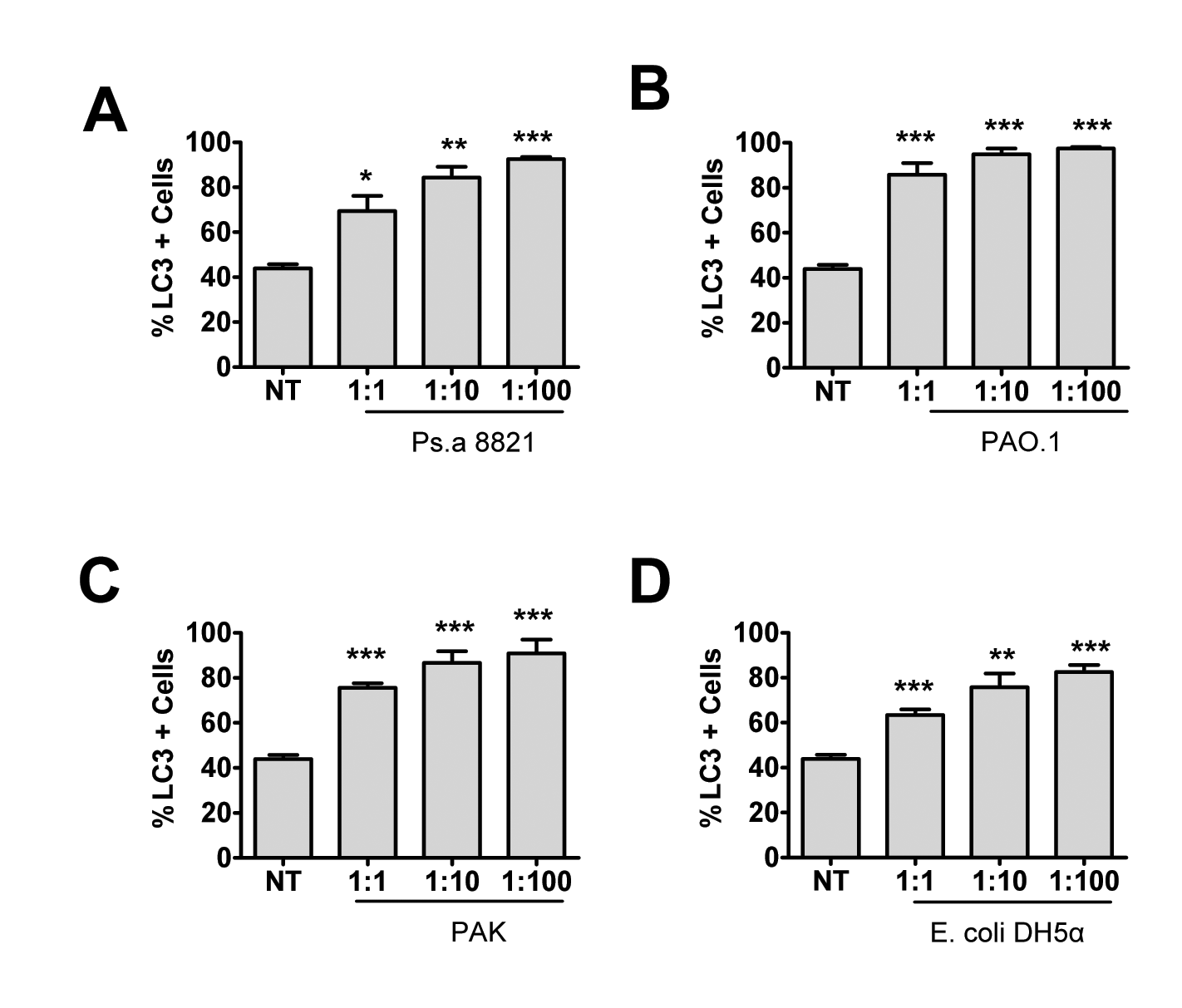

Supplement: Figure S1 — LC3 does not colocalize with granules in HMC-1 cells. HMC-1 5C6 cells were transiently transfected with LC3-GFP-mCherry then fixed and stained with toluidine blue 48 hours later. Cells were examined by fluorescence and light microscopy then LC3-mCherry positive puncta, and toluidine blue positive granules were identified. Colocalization of LC3 puncta (indicated with arrows) with mast cell granules was assessed (A). The average number of total granules and LC3 positive granules per cell was determined (B) (n = 100± SEM, ***p<0.005). The correlation between the number of granules and the number of LC3 positive puncta in each cell was also assessed (C) (n = 100). (TIF) [file pone.0072263.s001.tif]

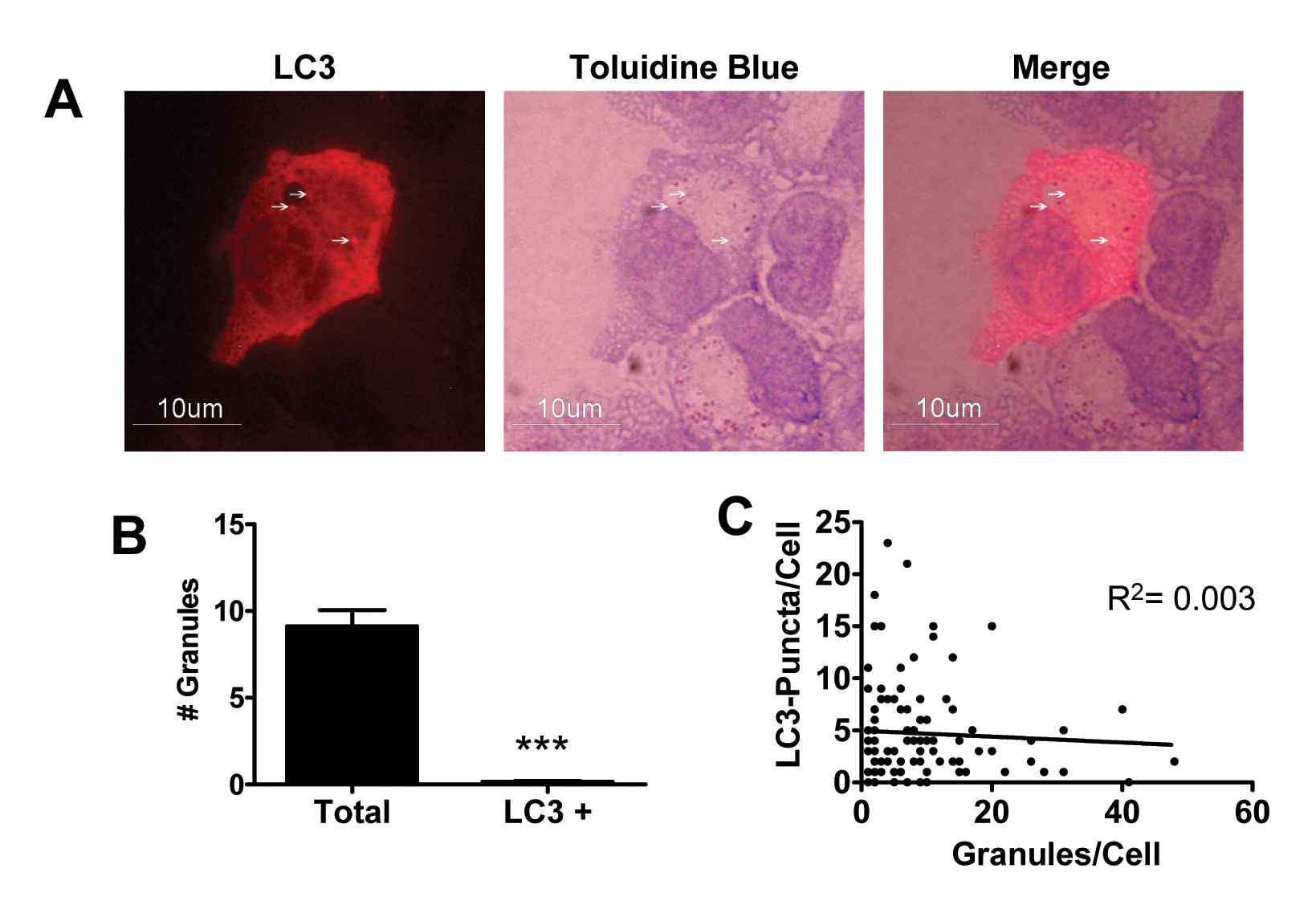

Supplement: Figure S2 — Strain and dose effects of P. aeruginosa and E. coli on autophagy in mast cells. Wild-type or LC3-GFP expressing HMC-1 5C6 cells were left untreated or infected at a 1∶1, 1∶10 and 1∶100 MOI with P. aeruginosa strains 8821 (A, E), PAO.1 (B, F), PAK (C, G) or E. coli strain DH5α (D, H). Cells were incubated for 18 hours at 37°C then lysed for Western blot analysis of LC3 and actin (A–D) or fixed and examined by fluorescence microscopy for the percentage of cells containing greater than 5 LC3-GFP puncta (E-H) (n = 3± SEM, *p<0.05, **p<0.01, ***p<0.005). (TIF) [file pone.0072263.s002.tif]

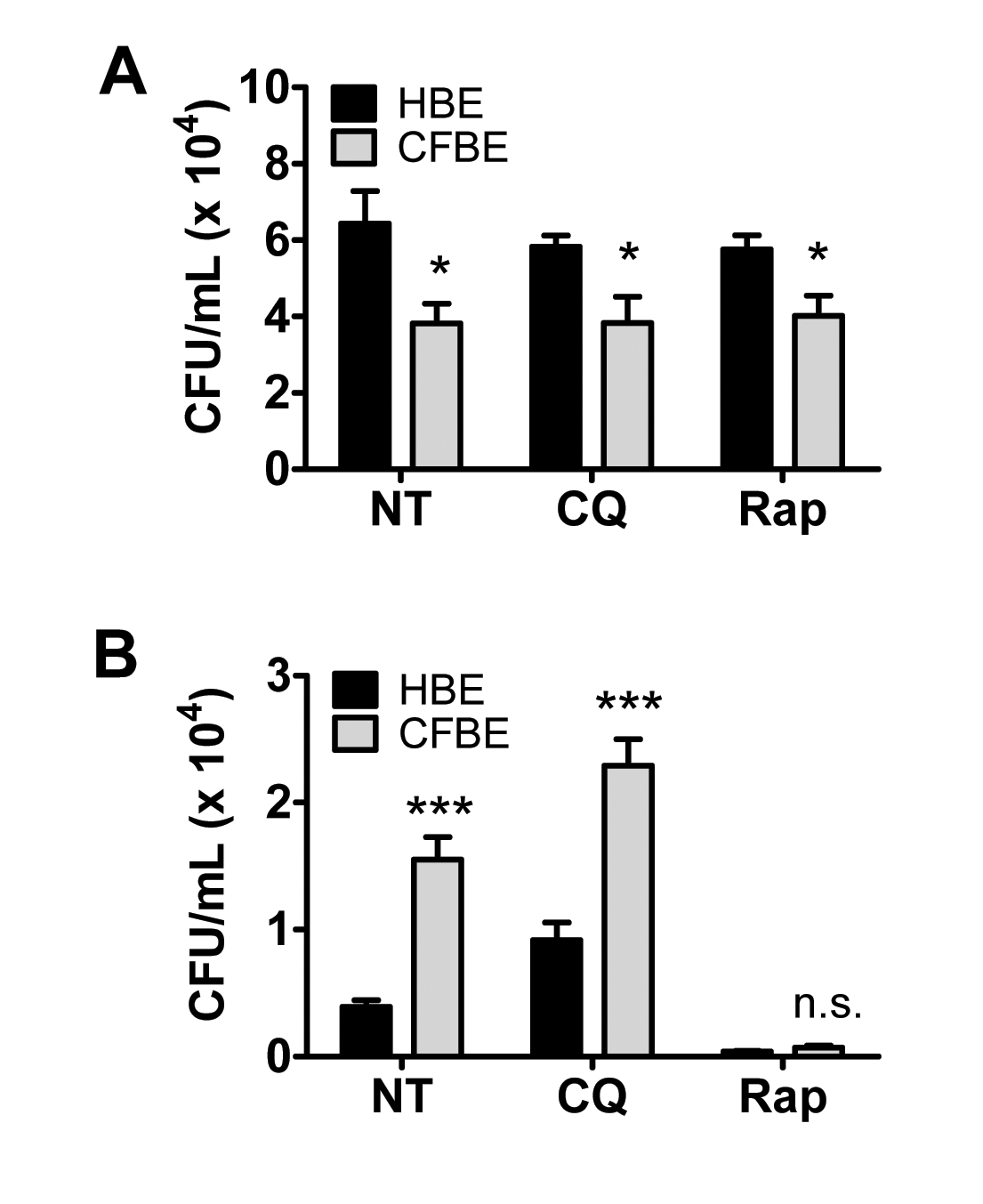

Supplement: Figure S3 — Induction of autophagy restores bacterial clearance in CF epithelial cells. Ten thousand 16HBE14o- (HBE) normal human bronchial epithelial cells or CFBE41o- (CFBE) homozygous CFTR ΔF508 cystic fibrosis bronchial epithelial cells were left untreated (NT) or pretreated for one hour with 20 µM chloroquine (CQ) or 2 µM rapamycin (Rap). Cells were then infected at a 1∶20 MOI with P. aeruginosa strain 8821 for 3 hours. Cell impermeable antibiotics were added for 10 minutes (A) or 3 hours (B) then serial dilutions of cell lysates were streaked to assess intracellular CFUs. The 10 minute and 3 hour time points represent bacterial internalization and bacterial killing respectively. (TIF) [file pone.0072263.s003.tif]

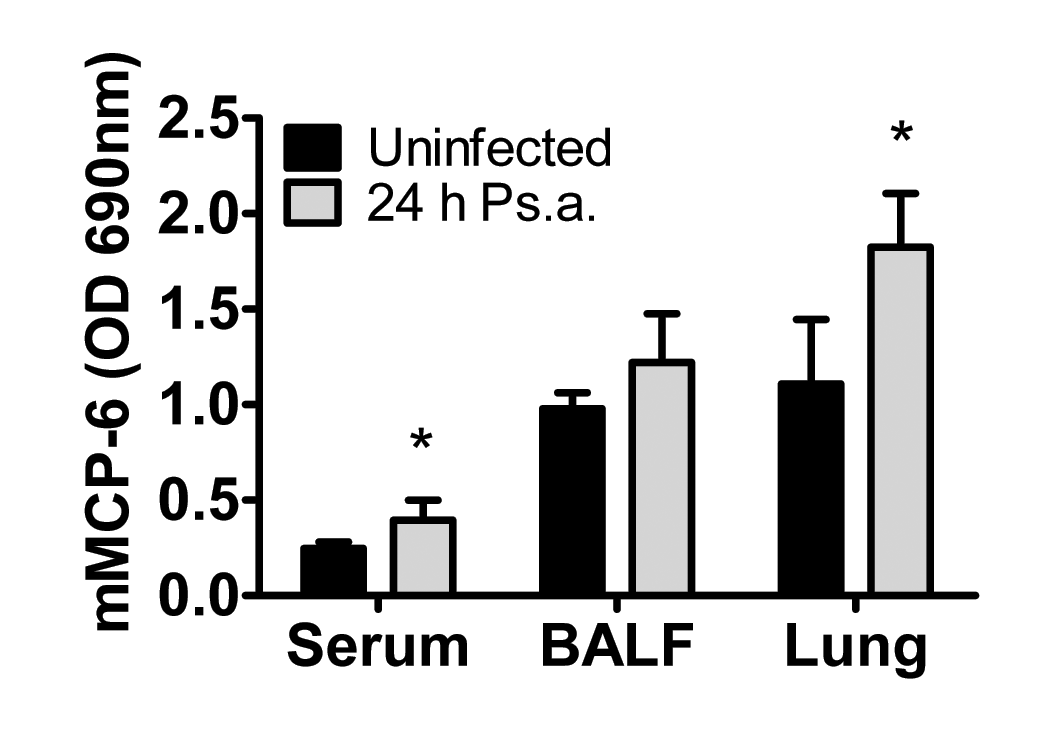

Supplement: Figure S4 — Mast cells contribute to host defense against P. aeruginosa. C57BL/6 mice were left uninfected, or infected intranasally with 109 P. aeruginosa strain 8821. Twenty four hours later serum, BALF and lung tissue was collected and the relative concentrations of the mast cell specific protease mMCP6 were determined by solid phase ELISA (n = 6 ± SEM, *p<0.05). (TIF) [file pone.0072263.s004.tif]

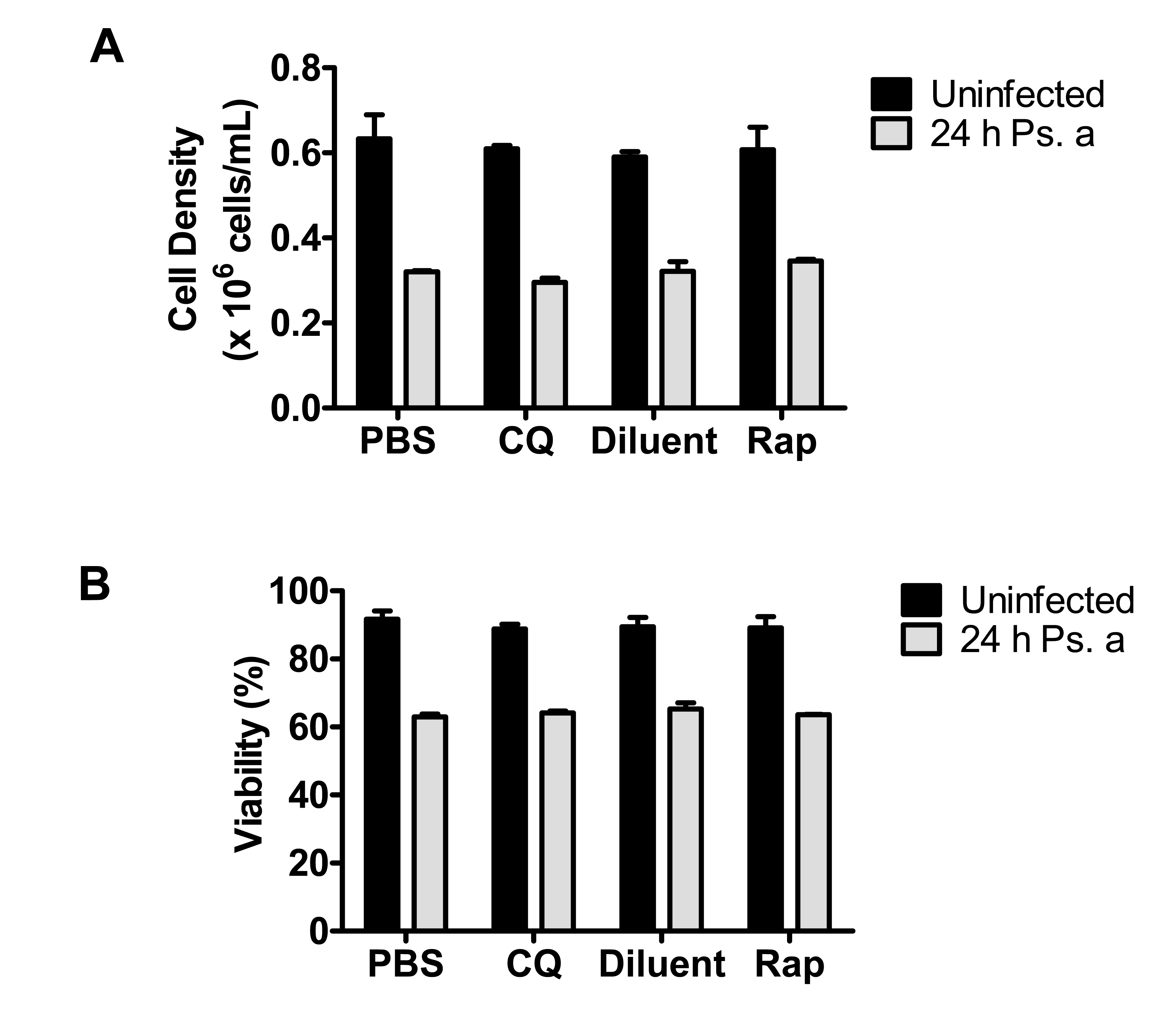

Supplement: Figure S5 — Pharmacological manipulation of autophagy does not effect mast cell survival following P. aeruginosa infection. BMMCs from C57BL/6 mice were pretreated for 24 hours with 20 µM chloroquine (CQ), 2 µM rapamycin (Rap), or an equivalent volume of PBS or rapamycin diluent. Cells were then infected with P. aeruginosa strain 8821 at an MOI of 1∶10 for 24 hours after which cell density (A) and viability (B) was determined by trypan blue staining (n = 3± SEM). (TIF) [file pone.0072263.s005.tif]

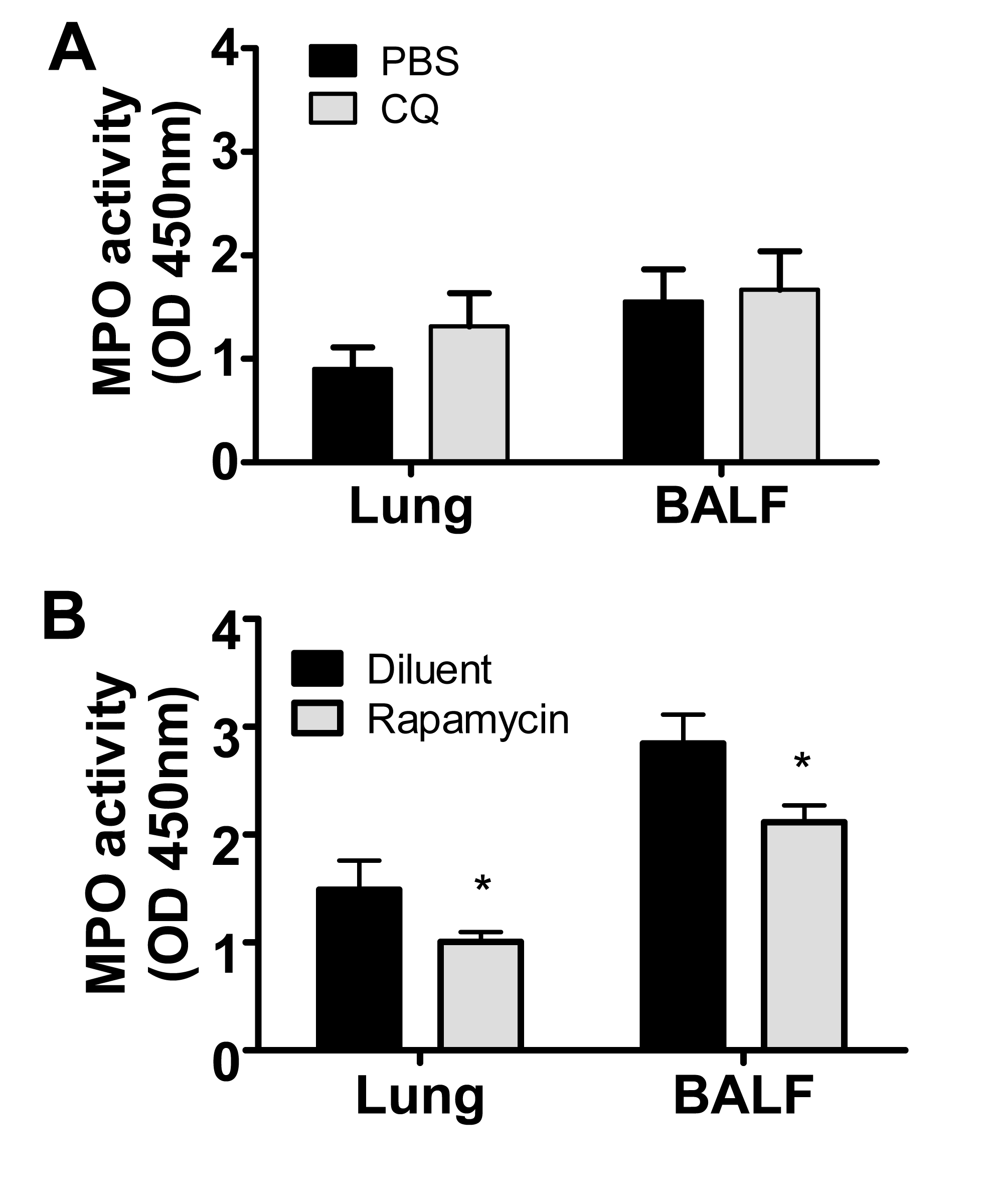

Supplement: Figure S6 — Rapamycin but not chloroquine impairs neutrophil infiltration into the lungs and BALF during P. aeruginosa lung infection. C57BL/6 mice were treated with intraparteneal injections of PBS or 60 mg/kg/day chloroquine (CQ) in PBS for 3 days (A) or with intraparteneal injections of diluent or 10 mg/kg/day rapamycin for 1 day (B), then infected intranasally with 109 CFU/mouse P. aeruginosa strain 8821. Twenty four hours later mice were sacrificed and lung tissue and BALF was assayed for the activity of the neutrophil specific enzyme MPO (n = 13-15± SEM, *p<0.05). (TIF) [file pone.0072263.s006.tif]
