# Supplementary material for: Autophagy Enhances Bacterial Clearance during P. aeruginosa Lung Infection
Source: PLoS One. 2013 Aug 28;8(8):e72263. doi: 10.1371/journal.pone.0072263 (PMC3756076; doi:10.1371/journal.pone.0072263)
Supplement: Table S1 — C57BL/6 mice were treated with intraperitoneal injections of PBS or 60 mg/kg/day chloroquine (CQ) in PBS for 3 days, and then left uninfected, or infected intranasally with 109 CFU/mouse P. aeruginosa strain 8821. Twenty four hours later mice were sacrificed and lung tissue and BALF was collected and assayed for concentrations of indicated cytokines via ELISA. (DOC) [file pone.0072263.s007.doc]

**Table S1: Chloroquine treatment does not affect inflammatory cytokine production following *P. aeruginosa* lung infection.**

|  |  | Uninfected^A^ | | 24 h *P. aeruginosa*^B^ | |
| --- | --- | --- | --- | --- | --- |
|  |  | PBS | Chloroquine | PBS | Chloroquine |
| Lung |  |  |  |  |  |
|  | IL-6 | 113.8 ± 10.8 | 103.7 ± 7.6 | 449.2 ± 60.7 | 601.9 ± 121.1 |
|  | RANTES | 160.9 ± 15.2 | 127.1 ± 8.9 | 8301.2 ± 1514.2 | 9363.1 ± 1500.1 |
|  | IL-1β | 208.9 ± 20.9 | 168.5 ± 25.2 | 501.0 ± 24.3 | 526.8 ± 8.9 |
|  | TNF | 156.3 ± 11.9 | 135.4 ± 18.9 | 721.7 ± 72.9 | 750.9 ± 57.9 |
|  | MIP-2 | 313.9 ± 19.3 | 333.9 ± 20.8 | 512.0 ± 34.8 | 558.1 ± 49.4 |
| BALF |  |  |  |  |  |
|  | IL-6 | 151.3 ± 10.8 | 155.4 ± 11.7 | 356.8 ± 78.5 | 304.7 ± 26.9 |
|  | RANTES | 2114.8 ± 463.2 | 2451 ± 441.1 | 4882.5 ± 888.0 | 6029.1 ± 10.20.1 |
|  | IL-1β | 523.4 ± 36.2 | 427.4 ± 40.9 | 4125.4 ± 493.5 | 4237.3 ± 1288.1 |
|  | TNF | 156.3 ± 11.9 | 142.4 ± 9.4 | 245.8 ± 21.6 | 245.1 ± 24.5 |
|  | MIP-2 | 381.1 ±15.7 | 256.0 ± 18.5 | 683.7 ± 32.0 | 710.6 ±25.5 |

^A^Data are the mean ± SEM of 5 mice per group (pg/mL)

^B^Data are the mean ± SEM of 13-14 mice per group (pg/mL)

**Table S1:** C57BL/6 mice were treated with intraperitoneal injections of PBS or 60 mg/kg/day chloroquine (CQ) in PBS for 3 days, and then left uninfected, or infected intranasally with 10^9^ CFU/mouse *P. aeruginosa* strain 8821. Twenty four hours later mice were sacrificed and lung tissue and BALF was collected and assayed for concentrations of indicated cytokines via ELISA.
