# Supplementary material for: Autophagy Enhances Bacterial Clearance during P. aeruginosa Lung Infection
Source: PLoS One. 2013 Aug 28;8(8):e72263. doi: 10.1371/journal.pone.0072263 (PMC3756076; doi:10.1371/journal.pone.0072263)
Supplement: Table S2 — C57BL/6 mice were treated with intraperitoneal injections of diluent or 10 mg/kg/day rapamycin for 1 day, and then left uninfected, or infected intranasally with 109 CFU/mouse P. aeruginosa strain 8821. Twenty four hours later mice were sacrificed and lung tissue and BALF was collected and assayed for concentrations of indicated cytokines via ELISA. (DOC) [file pone.0072263.s008.doc]

**Table S2. Rapamycin impairs inflammatory cytokine responses following *P. aeruginosa* lung infection**

|  |  | Uninfected^A^ | | 24 h *P. aeruginosa*^B^ | |
| --- | --- | --- | --- | --- | --- |
|  |  | Diluent | Rapamycin | Diluent | Rapamycin |
| Lung |  |  |  |  |  |
|  | IL-6 | 113.8 ± 10.8 | 103.7 ± 7.6 | 203.4 ± 17.2 | 180.9 ± 26.5 |
|  | RANTES | 127.1 ± 8.9 | 100.9 ± 20.3 | 1969.4 ± 162.8 | 1231.9 ± 159.3** |
|  | IL-1β | 208.8 ± 20.9 | 168.5 ± 25.2 | 2307.3 ±297.0 | 1309.9 ± 135.3*** |
|  | TNF | 120.3 ± 4.8 | 88.9 ± 6.9* | 201.8 ± 16.0 | 134.0 ± 10.5*** |
|  | MIP-2 | 333.9 ± 20.8 | 168.5 ± 25.17** | 1520.3 ± 117.1 | 1012.9 ± 173.4*** |
| BALF |  |  |  |  |  |
|  | IL-6 | 151.3 ± 10.8 | 155.4 ± 11.7 | 1633.5 ±138.2 | 1127.4 ± 165.4* |
|  | RANTES | 914.8 ± 159.3 | 971.6 ± 101.7 | 2726.3 ± 197.2 | 2250.7 ± 203.7 |
|  | IL-1β | 128.9 ± 5.01 | 80.4 ± 11.2** | 194.7 ± 29.9 | 159.6 ± 13.1 |
|  | TNF | 120.3 ± 4.7 | 88.8 ± 6.9** | 1283.8 ± 231.6 | 451.7 ± 55.3** |
|  | MIP-2 | 333.9 ± 20.7 | 168.5 ± 25.2*** | 826.1 ± 182.9 | 346.7 ± 71.1** |

^A^Data are the mean ± SEM of 5 mice per group (pg/mL)

^B^Data are the mean ± SEM of 18-19 mice per group (pg/mL)

*p<0.05, **p<0.01, ***p<0.001

**Table S2.** C57BL/6 mice were treated with intraperitoneal injections of diluent or 10 mg/kg/day rapamycin for 1 day, and then left uninfected, or infected intranasally with 10^9^ CFU/mouse *P. aeruginosa* strain 8821. Twenty four hours later mice were sacrificed and lung tissue and BALF was collected and assayed for concentrations of indicated cytokines via ELISA.
